# Enhancing the training of community engagement officers to address vaccine hesitancy: a university and local authority collaboration

**DOI:** 10.1177/17579139221145616

**Published:** 2023-08-17

**Authors:** S Murdan, N Ali, J Darlow, E Christopher, F Tolani, D Ashiru-Oredope

**Affiliations:** UCL School of Pharmacy, University College London 29-39 Brunswick Square, London WC1N 1AX, UK; UCL School of Pharmacy, London, UK; Bedford Borough, Central Bedfordshire and Milton Keynes Council’s Shared Public Health Services, Bedford, UK; Bedford Borough, Central Bedfordshire and Milton Keynes Council’s Shared Public Health Services, Bedford, UK; Bedford Borough, Central Bedfordshire and Milton Keynes Council’s Shared Public Health Services, Bedford, UK; UCL School of Pharmacy, London, UK; UK Health Security Agency, London, UK

## Introduction

Vaccine hesitancy/scepticism remains an issue, and ongoing actions to promote vaccination are needed. While no single intervention strategy addresses all instances of vaccine hesitancy, effective methods have been identified. For example, recommendations from a healthcare professional and dialogue-based, directly targeted approaches with personalised and tailored communications for different audiences, including from a trusted community member.^1–6^

## University and Local Government Joining Forces to Increase Their Impact

In mid-2020, the UCL School of Pharmacy started training Pharmacy undergraduate students to become Vaccination Champions and promote vaccination in their multitude of identities, that is, not only as a healthcare students but also as a family and community member, neighbour, friend, etc.^7^ We have also provided resources for Pharmacy professionals.^8^

In response to COVID-19, the Bedford Borough, Central Bedfordshire and Milton Keynes Council’s shared Public Health Service employed place-based teams of four COVID-19 and Health Inequality Community Engagement Officers. As part of their role within the wider Health Protection and Disadvantaged Groups team, the Community Engagement Officers visit and engage with places of worship, key businesses and community groups to develop relationships with local residents. By building relationships with religious and community leaders, trusted individuals and Local Councillors, the teams are able to determine barriers to the uptake of COVID-19 vaccines. They can also answer questions and provide information and solutions, such as dispelling myths and misinformation or arranging taxis to vaccination centres.

In this article, we describe how the University and the local government teams worked together to maximise the impact of the Community Engagement Officers, in line with fulfilling Universities’ Third Mission,^9,10^ with the priority herein being the transfer of academic knowledge to help resolve a societal challenge.

**Figure:**
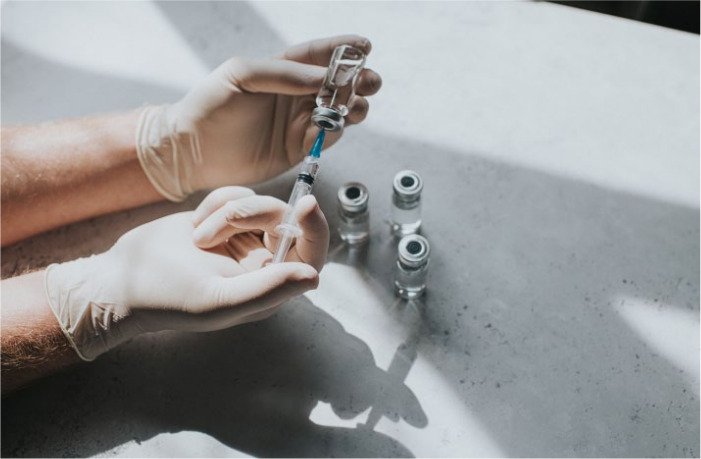


## How it Started

The academic team approached Bedford Borough Council to offer support with addressing vaccine hesitancy, given their experience of training Pharmacy students to become Vaccination Champions. Following consultation with Public Health’s Health Protection and Disadvantaged Groups team, it was agreed that the academic partners would deliver, at no cost, a 2 h live online workshop to the Community Engagement Officers (adapting the resources previously used with students), conduct pre and post workshop surveys, hold a follow-up session and publish a report about the joint activity. The Public Health team suggested topics and questions relevant to their residents and strategic direction for which they required more information.

The aim of the joint activity was to increase Community Engagement Officers’ knowledge about COVID-19 vaccines and vaccination, and their confidence when engaging with vaccine-hesitant individuals.

## Delivering the Workshop

The workshop surveys of participants indicated a greater proportion of female participants, a range of ages (20–59 years), ethnicities (Black or Black British – African, Caribbean, White British, White Irish, White any other background, Asian or Asian British (Indian/any other Asian background) and Mixed) and backgrounds in Science/Health (from none to a Master’s degree in a health-related subject and experience in vaccine community engagement). Answers about vaccination showed some gaps in knowledge, while one participant asked for more information about dealing with anti-vaxxers and the best way to engage with young people. The 2 h online live workshop was delivered to 13 Community Engagement Officers, and four members of the wider Health Protection and Disadvantaged Groups team. The workshop was conducted using Microsoft (MS) Teams and consisted of presentations, breakout rooms and a question-and-answer session. Presentations covered frequently encountered topics, including vaccine efficacy and effectiveness, the waning of effectiveness and the need for booster doses, adverse effects, vaccination in pregnancy and herd immunity. They also looked at the vaccine development process and factors which sped up the development of COVID-19 vaccines, hesitancy and barriers to vaccine uptake, COVID-19 vaccine misconceptions and ‘Dos and Don’ts’ when addressing vaccine hesitancy.

The first breakout room slot took place prior to the related presentation and engaged participants in a discussion on the common misconceptions, concerns and barriers to vaccination they had encountered. Breakout room rapporteurs reported collective encounters of concerns about infertility, needle phobias, denial, a belief in their immune system looking after them, getting COVID-19 with the booster dose, adverse effects and the impact on work, blood clots, taking the vaccine while pregnant, and concerns about access and peer/family pressure.

A second breakout room slot, which took place towards the end of the workshop, resulted in Community Engagement Officers reporting a sense of being more ‘clued-up’. In particular, that they now had more facts and scientific knowledge to ‘back up’ their conversations, and that the training had increased confidence in their answers, and a commitment to listen more to people to understand ‘where they are coming from’.

## Post Workshop Feedback and Case Study

The post workshop survey showed very positive feedback about the workshop overall. Participants found the session stimulating, interesting and relevant to their role; the session was paced well and the duration was appropriate, and participants complimented the speakers’ audibility and explanation, and adequacy and preparedness of slides. An example of a comment:

*The workshop was really engaging, the slides were visual and relevant, explaining complex scientific data and knowledge clearly. The breakout session worked really well and I think the group discussions (although maybe some were reluctant to contribute) were very effective and support knowledge sharing and learning through experiences. My only point of change would be to allow for a bit more time, so maybe the session should be 3 hours with two set–breaks (cover that in the intro – so layout the timing of the whole workshop).*

Within a few weeks of the workshop, participants submitted a case study of an interaction during their work, where they had used the training. Several participants had used the diagrams provided in the slides to communicate more effectively, for example, to explain the speed of the COVID-19 vaccine development and the potential for COVID-19 infection post vaccination as the vaccine only reaches full efficacy after 2–3 weeks. Other participants reported providing information about and supporting the use of the free taxi service to vaccination centres and about dealing with needle phobia. One participant explained how useful the provided information would have been during their own family’s experience of COVID-19, while another used their knowledge to convert technical documents on the effectiveness of vaccination against long COVID-19 into digestible information for the public. Several participants reported conversations with individuals who were totally against vaccination, resulting in an inability to promote vaccination.

## Post Workshop Follow-Up Session

Ten weeks after the online workshop, Community Engagement Officers attended an online follow-up session to share their experiences. During the session, they reported that during their interactions, they could explain things more clearly, for example, the speed of COVID-19 vaccine development, using the relevant workshop slides and felt

*more secure in what I was saying and more confident in the knowledge that I’d been given*,

*had the statistics to back it up*,

*more strongly and passionate about what I was doing*,

and that their interlocutors *understood it* and *accepted it a lot better* and went away with information leaflets:

*It is like we can win them*

Community Engagement Officers still encountered vaccine scepticism and public mistrust in the government from some. It is noteworthy that race and religion were frequently mentioned in vaccine conversations. It is therefore essential for anyone promoting vaccination to have a working knowledge of different faith and ethnic groups, in addition to a scientific understanding of vaccines and vaccination.

Participants who completed the whole course were awarded a Vaccination Champion Certificate by the Local Authority (template in Supplementary Info).

## The Sequel

The impact and importance of Community Engagement Officers as a permanent fixture within local authority Public Health has again been demonstrated to funders, and the teams involved in this collaborative training project will continue in their roles until the end of March 2023. As they move forward, ‘Living with COVID’, ‘Making Every Contact Count’, and the wider Health Protection remit including screening and immunisation programmes impacted by COVID are on the agenda.

## Conclusion

We hope that by reporting this collaboration, we inspire others to engage in similar activities, as these can have large impacts at fairly low costs to all partners. By adapting teaching and learning resources that had previously been used with undergraduates, academic cost was lowered, making the collaboration more feasible. The academic team has a greater understanding of the reality on the ground, and both parties have gained new perspectives of community engagement.
